# A Novel Unsupervised Segmentation Quality Evaluation Method for Remote Sensing Images

**DOI:** 10.3390/s17102427

**Published:** 2017-10-24

**Authors:** Han Gao, Yunwei Tang, Linhai Jing, Hui Li, Haifeng Ding

**Affiliations:** 1 Key Laboratory of Digital Earth Science, Institute of Remote Sensing and Digital Earth, Chinese Academy of Sciences, Beijing 100094, China; gaohan@radi.ac.cn (H.G.); lihui@radi.ac.cn (H.L.); dinghf@radi.ac.cn (H.D.); 2 University of Chinese Academy of Sciences, Beijing 100049, China

**Keywords:** high spatial resolution remote sensing, image segmentation, unsupervised segmentation evaluation, spatial stratified heterogeneity, statistical features

## Abstract

The segmentation of a high spatial resolution remote sensing image is a critical step in geographic object-based image analysis (GEOBIA). Evaluating the performance of segmentation without ground truth data, i.e., unsupervised evaluation, is important for the comparison of segmentation algorithms and the automatic selection of optimal parameters. This unsupervised strategy currently faces several challenges in practice, such as difficulties in designing effective indicators and limitations of the spectral values in the feature representation. This study proposes a novel unsupervised evaluation method to quantitatively measure the quality of segmentation results to overcome these problems. In this method, multiple spectral and spatial features of images are first extracted simultaneously and then integrated into a feature set to improve the quality of the feature representation of ground objects. The indicators designed for spatial stratified heterogeneity and spatial autocorrelation are included to estimate the properties of the segments in this integrated feature set. These two indicators are then combined into a global assessment metric as the final quality score. The trade-offs of the combined indicators are accounted for using a strategy based on the Mahalanobis distance, which can be exhibited geometrically. The method is tested on two segmentation algorithms and three testing images. The proposed method is compared with two existing unsupervised methods and a supervised method to confirm its capabilities. Through comparison and visual analysis, the results verified the effectiveness of the proposed method and demonstrated the reliability and improvements of this method with respect to other methods.

## 1. Introduction

The ultimate goal of remote sensing is to mirror, elucidate, quantify, and describe surface patterns to contribute to the understanding of the underlying phenomena and processes [1]. In recent years, very high spatial resolution (VHR) earth observation images obtained from satellite and airborne sensors have become increasingly available and have provided more detailed spatial structures and textural features of ground objects. Geographic object-based image analysis (GEOBIA) is a widely used and particularly effective method for the analysis of VHR images that overcomes the limitations of pixel-based image analysis. GEOBIA groups spatially adjacent pixels into spectrally homogenous image objects using a segmentation rule or criterion that keeps the within-object spectral variation small. Moreover, GEOBIA can use the spectrally homogeneous segments of images to effectively incorporate the spectral and spatial information of objects as features that assist with additional tasks such as photointerpretation, recognition or classification [2,3,4,5,6].

Partitioning an image into spatially contiguous and relatively homogeneous regions, also known as image objects or segments, is a key step of the GEOBIA approach [1,7]. The resulting segments, or image objects, are then used in the following image analysis (e.g., object-based classification), and the quality of the segmentation results explicitly affects the accuracy and reliability of the workflow. Ideally, these image objects that are composed of similar spectral values should possess intrinsic size, shape, and geographic relationships with the real-world scene they represent [4]. They should have distinct boundaries, be relatively coherent and representative of the real-world objects. However, both analysis and practice show that the segmentation quality of remote sensing images varies according to different land cover types and applied segmentation algorithms [8,9,10]. In addition, setting different parameters (e.g., segmentation scale) of a certain segmentation algorithm can also result in differently segmented images. Thus, evaluating the performance of segmentation algorithms is important to identify effective segmentation algorithms or optimally parameterize a particular segmentation algorithm [9,11].

The existing strategies for evaluating segmentation quality can be divided into five categories: subjective evaluations, system-level methods, analytical methods, supervised evaluation methods, and unsupervised evaluation methods [12]. The most commonly used method is a subjective evaluation by human observers. However, this visual or qualitative evaluation is impractical for processing remote sensing images because it is commonly practiced at the expense of time and labor, and involves different interpreters that are inherently subjective. System-level evaluation methods examine the impact of segmentation algorithms based on the empirical system results (e.g., the final classification accuracy reflects the previous segmentation quality in an object-based classification process), which is indirect and dependent on specific applications. Without experiments, analytical methods treat segmentation algorithms directly by considering some measures (e.g., complexity), which are assumed to be appropriate measures of a priori knowledge, and thus are seldom used in isolation [13].

Supervised evaluation methods, also referred to as empirical discrepancy methods or relative evaluations methods, are designed to quantitatively measure the dissimilarity between segmented images and manually segmented reference images to assess the performance of segmentation algorithms. In other words, these methods attempt to determine how different the segmented image is from the ground truth, which is derived from expert interpretation [11,14]. Several effective and quantitative supervised methods have been developed and tested [14,15,16,17,18,19,20,21,22,23]. However, different experts will subjectively ascribe different interpreters to the references created by them [15]. Moreover, manually generating objective reference images for large-scale high spatial resolution remote sensing images is also a difficult and time-consuming task.

Unsupervised evaluation methods, also referred to as empirical goodness methods or stand-alone evaluation methods, evaluate the performance of segmentation algorithms without the need of reference images. Unsupervised evaluation judges segmented images with certain quality criteria that were established according to human perception [24]. As the name implies, the most significant advantage of unsupervised methods is the ability to assess segmentations independently of a manually created reference image and use criteria that enable the quantification of the quality of segmentation results without any a priori knowledge. Most evaluation criteria are based on statistical measures such as gray-level standard deviation or the disparity of each object region or class in the segmentation results, which can be computed automatically in real time, making it possible for self-tuning and the dynamic adjustment of the parameters for a better result [25]. One widely accepted benchmark of what constitutes a good segmentation has been defined: (1) regions should be uniform and homogeneous with respect to some characteristics; (2) adjacent regions should have significant differences with respect to the characteristics in which they are uniform; (3) region interiors should be simple and without holes; and (4) boundaries should be simple, not ragged, and spatially accurate [26]. The first two conditions are defined as characteristic criteria, and the last two are defined as perceptual or semantic criteria. However, for highly textured and natural images, such as VHR remote sensing images, only the characteristic criteria can realistically be applied [12]. Building on this point, a good segmentation method should maximize the intra-segment homogeneity and inter-segment heterogeneity to satisfy the principles of the characteristic criteria.

Many unsupervised approaches for remote sensing images have been presented in the literature. Stein and De Beurs used complexity metrics to quantify the semantic accuracy of image segmentations of two Landsat images [27]. Chabrier et al. tested and compared six different evaluation criteria and three different algorithms for segmenting radar and multispectral aerial images [24]. Espindola et al. measured intra-segment homogeneity using the weighted variance of the near-infrared (NIR) band and measured intra-segment heterogeneity using a spatial autocorrelation measure, global Moran’s I, for the NIR band as well [28]. Kim et al. computed unweighted variance and global Moran’s I and graphed each separately for evaluation [29,30]. Radoux and Defourny used a combination of normalized post-segmentation standard deviation and border discrepancy to evaluate the segmentation results in a rural area [31]. Faur et al. defined the mean square error as the distortion function and obtained the optimal number of clusters based on rate-distortion theory [32]. Corcoran et al. proposed an evaluation metric that considered spatial properties [33]. Zhang et al. proposed the use of weighted combination using a modified color uniformity to indicate homogeneity and the normalized variance of a mean feature vector of all regions to indicate heterogeneity [34]. Troya et al. proposed an unsupervised local metric named under-and over-segmentation aware (UOA) metric, which evaluates the quality of each segment individually as a function of its spatial neighborhood and a given homogeneity criterion [35]. Shi et al. proposed a novel unsupervised measure mainly based on objectness which reflects an essential attribute of an object. The measure considers the quantity distortion as an additional constraint for the single object segmentation [36]. Bock et al. proposed a modification of the method in [28] to mitigate some existing problems by an alternative normalization scheme [37].

In previous studies, the global intra- and inter-segment indicators were combined to assign an overall goodness score to the segmentation in most unsupervised evaluation methods. However, the design of effective indicators, reasonable combination strategies, and operation domains remain challenging for unsupervised methods. This study proposes a novel unsupervised evaluation method that can overcome these difficulties. Based on characteristic criteria, spatial stratified heterogeneity and spatial autocorrelation serve as the indicators that are calculated on a spectral-spatial feature set. These indicators are then combined into a single overall evaluation metric for scoring and ranking multiple segmented images. Different from previous studies, this paper makes the following contributions: (1) it captures a feature set to describe image objects in both the spectral and spatial domains; (2) designs a new composite evaluation metric that accounts for spatial stratified heterogeneity to measure intra-segments; (3) devises a reasonable indicator combination strategy based on the Mahalanobis distance; and (4) validates the evaluation method using results from multiple segmentation algorithms targeting different types of landscapes.

The remainder of the paper is organized as follows: Section 2 provides a detailed discussion of the evaluation methods and segmentation algorithms employed in this study. The experimental results are presented in Section 3, and the effectiveness and advantages of the proposed method are revealed. Further analysis of these results is discussed in Section 4. Finally, conclusions are drawn in Section 5.

## 2. Methods

**Figure 1:**
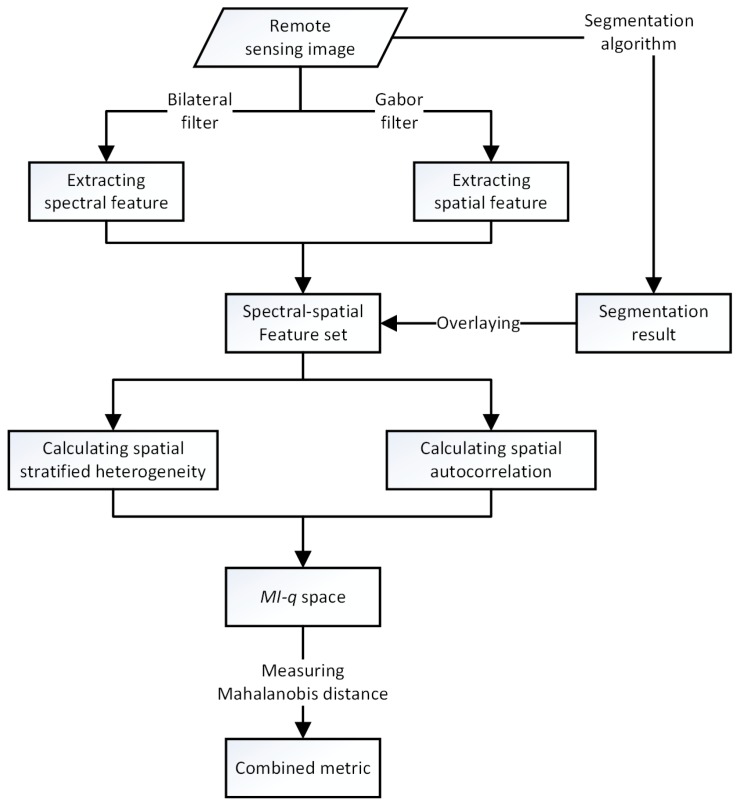
Flowchart of the proposed method to evaluate segmentation quality.

An optimal image segmentation result is supposed to satisfy the principle that maximizes intra-segment homogeneity and inter-segment heterogeneity. According to the characteristic criteria of unsupervised evaluation methods discussed in Section 1, a schematic of the calculation of global intra- and inter-segment “goodness measures” on a spectral-spatial feature set to evaluate segmentation quality is shown in Figure 1. First, a spectral-spatial feature set is captured and stacked from a raw remote sensing image. Then, the spatial stratified heterogeneity and the spatial autocorrelation of the segmentation results are measured as the indicators of the feature set. Finally, these two indicators are combined into a single metric to reveal the segmentation quality.

### 2.1. Extraction of Feature Set

**Figure 2:**
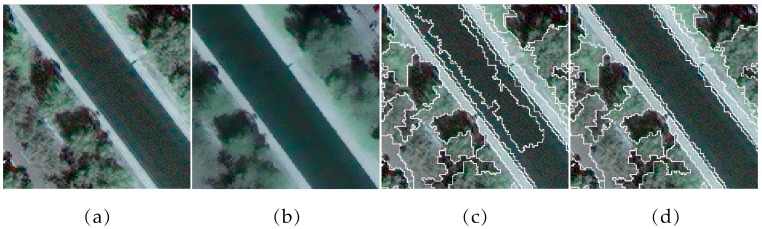
(**a**) The raw sample image; (**b**) the spectral feature image extracted from (**a**) using the bilateral filter ($\sigma_{s}=3$; $\sigma_{r}=0.1)$; (**c**) the segmentation results of image (**a**) at scale 50; and (**d**) the segmentation results of image (**a**) at scale 55. The object boundaries are delineated by the white line.

The majority of the existing unsupervised evaluation methods in the literature attempt to use the original spectral features as the basis for calculation, which is effective if each primitive object has a uniform spectrum [33]. However, VHR remote sensing images feature high spatial complexity, and ground objects are typically characterized by a combination of spectral and textural properties. The texture of remote sensing images is a reflection of the spatial characteristics of the ground objects. Surface objects in images contain small-scale textures that are sensitive to segmentation algorithms, such as the water surface of the image in Figure 2a and the resulting segmentation results in Figure 2c,d. It is difficult to accurately represent these real objects using only the original spectral features without spatial features. In addition, the quality of the evaluations performed on the original images will lead to the misinterpretation of the segmentation results given the real form of the objects. Hence, it is necessary to extract a feature set composed of reasonable spectral and spatial features to represent the ground objects in remote sensing images.

#### 2.1.1. Spectral Feature Extraction

The feature representation should be a good fit to the human visual perception of the objects. Given within-object variation or a complicated texture, the original intensity values from remote sensing images are not suitable for direct use as spectral features. A bilateral filter is applied to extract the spectral feature to remove such within-object variation while maintaining the boundary information. The bilateral filter is a non-linear, edge-preserving and noise-reducing filter for images that can smooth an input image while preserving its edges. The bilateral filtering kernel W is given by the following expression [38]:

(1) $$W_{ij}\left(I\right)=\frac{1}{K_{i}}exp\left(-\frac{{\left|x_{i}-x_{j}\right|}^{2}}{\sigma_{s}^{2}}\right)\cdot exp\left(-\frac{{\left|I_{i}-I_{j}\right|}^{2}}{\sigma_{r}^{2}}\right),$$

where *i* and *j* are pixel indexes of the raw image, *x* is the coordinate of the current pixel to be filtered, *I* is the intensity value and $K_{i}\;$is a normalizing parameter to ensure that $\underset{j}{\sum }W_{ij}=1.$ The parameters $\sigma_{s}\;$and $\sigma_{r}\;$represent the spatial similarity and the intensity of the similarity, respectively. Figure 2a,b shows that the water surface in the filtered image (Figure 2b) is more uniform than that in the raw image (Figure 2a).

In Figure 2, the main difference between results images in Figure 2c,d involves the segment boundaries of the water object that were generated using different segmentation scale parameters from image Figure 2a . Segmentation result in Figure 2d clearly describes the water surface more accurately, while Figure 2c is over-segmented. However, the homogeneity of each water surface object in Figure 2c is higher than in Figure 2d, which could lead to metrics calculated on the image in Figure 2a indicating that the result in Figure 2c is superior if only the intra-segment properties are considered. Hence, if the metric is computed from Figure 2b, where the objects are more spectrally uniform than the raw image, this can enhance the homogeneity within the water object of the result in Figure 2d, thus guiding the metric to reflect the real forms of the image objects to a certain degree.

#### 2.1.2. Spatial Feature Extraction

**Figure 3:**
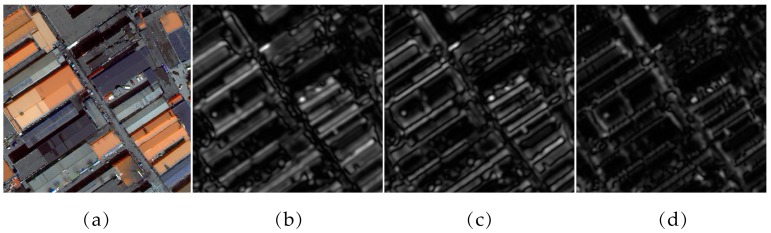
(**a**) The original sample image; (**b**) the response image with *v* = 2, *u* = 1; (**c**) the response image with *v* = 2, *u* = 1.5; and (**d**) the response image with *v* = 1, *u* = 1.5.

As discussed above, ground objects in VHR images can be described by spatial features, which should be exploited for the segmentation evaluation. Spatial features provide accurate localization of texture boundaries that can be used to adequately delineate the real forms of ground objects. This complementary information can be used to discriminate different ground objects that are spectrally similar. This paper implements the two-dimensional Gabor wavelet to characterize the spatial information. The Gabor wavelet decomposes an image in multiple scales and multiple orientations and is expressed as follows [39]:

(2) $$G_{u,v}\left(x,y\right)=\frac{∥k{∥}^{2}}{\sigma^{2}}\exp\left(-\frac{∥k{∥}^{2}\left(x^{2}+y^{2}\right)}{2\sigma^{2}}\right)\left[\exp\left(ik\cdot \left[\begin{array}{c} x\\ y \end{array}\right]-\exp\left(-\frac{\sigma^{2}}{2}\right)\right)\right],$$

(3) $$k=\left[\begin{array}{c} k_{x}\\ k_{y} \end{array}\right]=\left[\begin{array}{c} k_{v}cos\varphi_{u}\\ k_{v}sin\varphi_{u} \end{array}\right],$$

where $∥\cdot ∥\;\mathrm{denotes}\;\mathrm{the}\;\mathrm{norm}\;\mathrm{operator}\;;\;k_{v}=2^{-\frac{v+2}{2}}\pi ,\;\varphi_{u}=u\frac{\pi }{U},\;v\;\mathrm{and}\;u$ determine the center frequency and orientation, respectively; U  is the number of orientations; and σ is the ratio of the Gaussian window width to the wavelength. Applying a bank of Gabor filters with V frequencies and U orientations to a raw image can generate V×U response images, which contain the local energy changes of the raw image that are used as the spatial features for the subsequent analysis. In Figure 3b–d, three response images with two frequencies and orientations (U = 2) are presented as examples. The corresponding texture and edges of the raw image are extracted by a fixed set of Gabor filters.

In this paper, the bilateral spectral features and the Gabor spatial features are stacked into a joint spectral-spatial feature set, allowing all objects and boundaries to be described spectrally and spatially. This feature set, which is generated during this process, can be regarded as the base image, from which all statistics of the following evaluation indicators are computed to estimate the segmentation results.

### 2.2. Spatial Stratified Heterogeneity and Autocorrelation

Spatial heterogeneity and spatial autocorrelation are two main features of ecological and geographical phenomena. The spatial heterogeneity between strata or areas, which is composed of a number of units, is called the spatial stratified heterogeneity, which is reflected and visualized by the spatial stratification of the heterogeneity of the classification [40]. In principle, stratification of the heterogeneity partitions a target by minimizing the within-strata variance and maximizing the between-strata variance of an attribute. Similarly, this study regards the segmentation of a remote sensing image as the stratification of heterogeneity, where observations are homogeneous within each stratum (intra-segment) and are heterogeneous between strata (inter-segment). Stratified heterogeneity is most likely to be significant if the spectral values within the segments are homogeneous or the variances within the segments approach zero; the heterogeneity of a segmentation equals zero when there is no difference between the segments.

The spatial stratified heterogeneity of an attribute can be measured using the q-statistic in the geographical detector [41]. Specifically, an image composed of *N* pixels is segmented into *h* = 1, 2, …, *L* segments, where segment *h* consists of $N_{h}$ pixels; $Y_{i}$ and $Y_{h_{k}}$ represent the spectral values of pixel *i* in the feature set and the segment *h*, respectively. The mean value of the feature in segment *h* is ${\bar{Y}}_{h}=\left(\frac{1}{N_{h}}\right)\overset{N_{h}}{\underset{k}{\sum }}Y_{h_{k}}$ and the mean value of the whole feature set is $\bar{Y}=\frac{1}{N}\overset{N}{\underset{i}{\sum }}Y_{i}.$ Then, the q-statistic can be calculated as follows:

(4) $$q=1-\frac{\sum_{h=1}^{L}\sum_{k=1}^{N_{h}}{\left(Y_{h_{k}}-{\bar{Y}}_{h}\right)}^{2}}{\sum_{i=1}^{N}{\left(Y_{i}-\bar{Y}\right)}^{2}},\;$$

The q-statistic is a monotonic function of the strength of the spatial stratified heterogeneity. The q value is within [0, 1], and it increases as the strength of the intra-segment homogeneity increases. However, as the segmentation scale decreases (i.e., the number of image objects increases), the q value increases. In an extreme over-segmentation case where each pixel is a segment, the q value is 1. Therefore, it is difficult to use a single metric to evaluate the quality of the segmentation results.

Spatial autocorrelation statistics measure the spatial dependency among the observations in geographical space. The attributes of the fields or objects that are at closer geographical sites are more similar than those at distant sites, leading to spatial clusters or spatial dispersions, which can be used as a metric to assess the inter-segment global goodness. Weak spatial autocorrelation of a segmentation result indicates that the neighboring segments (i.e., segments sharing a boundary) are significantly different, suggesting high inter-segment heterogeneity. The Global Moran’s I (MI) is a measure used to estimate the degree of spatial autocorrelation between adjacent locations [42]. In previous studies, it has been proven that MI is an effective and reliable indicator of segmentation quality [10,28]. MI is calculated using the following formula:

(5) $$MI=\frac{L\sum_{h=1}^{L}\sum_{d=1}^{L}w_{hd}\left({\bar{Y}}_{h}-\bar{Y}\right)\left({\bar{Y}}_{d}-\bar{Y}\right)}{\left(\sum_{h=1}^{L}{\left({\bar{Y}}_{h}-\bar{Y}\right)}^{2}\right)\left(\sum_{h=1,h\neq d}^{L}\sum_{d=1}^{L}w_{hd}\right)},$$

where L is the number of segments indexed by h and d, $w_{hd}\;$ is an element of a matrix of spatial weights, and ${\bar{Y}}_{h}\;$ and $\bar{Y}\;$represent the mean spectral value of the feature set in segment h and the entire segmented image, respectively. The spatial weights matrix $w_{hd}$ reflect the geographic relationship between segments h and d and have many calculation methods. This study assigns a weight of 1 if segment h and segment d are neighbors, otherwise the weight is 0. MI ranges from −1 to 1, where negative values indicate negative spatial autocorrelation and positive values indicate positive spatial autocorrelation. Weak autocorrelation among segments in geographical space is desirable. Therefore, a local minimum of |MI|, the absolute value of MI, indicates that the segmentation result has a high inter-segment heterogeneity, which means a clear distinction between different segments.

### 2.3. Combination Strategy

Segmentation evaluation requires simultaneous measurements in both inter-segment disparity and intra-segment uniformity. Therefore, the MI and q values are jointly used to reveal segmentation quality, and they vary inversely as the quality changes. In an ideal case, a higher q value and a lower |MI| of one segmentation result indicate a relatively desirable segmentation quality. However, it is sometimes difficult to evaluate the quality using two separate measures concurrently. One solution is to combine the two measures into an integrated metric. This study presents an MI−q space measure that is a combination of MI and q values to visually indicate segmentation quality by geometric illustration. A segmentation result composed of a quality point with two variables corresponds to a quality point in this two-dimensional space. The worst segmentation cases occur at point (1,0) or point (−1,0) in the MI−q space. As mentioned above, the absolute values of MI are used in this combination strategy. Therefore, the quantitative value of the dissimilarity from the worst result (point (1,0)) can characterize the segmentation quality and make it comparable. A distance metric can be utilized to define the dissimilarity measure between the points.

The most commonly used distance metric is the Euclidean distance. However, the Euclidean distance is sensitive to the scales of the variables involved. The Euclidian distance assumes that each variable of the data point is equally important and all variables are measured in the same units. The scale problem that is inherent in Euclidean distance can be solved by defining dissimilarity measures with the Mahalanobis distance [43], which is unitless, scale-invariant and considers the covariance among the variables when calculating the distance. Therefore, the Mahalanobis distance can characterize the objective function as follows:

(6) $$d_{M}\left(X_{o},X_{s}\right)=\sqrt{{\left[X_{o}-X_{s}\right]}^{T}\cdot \Sigma^{-1}\cdot \left[X_{o}-X_{s}\right]},$$

where $X_{o}\;$is point (1,0); $X_{s}=\left({\left|MI\right|}_{s},q_{s}\right)$ is the quality point with two components, the |MI| and q values of the segmentation result *s*, *s =* {1,2,…,S}; S is the number of segmentation results; and Σ  is the covariance matrix of all quality points involved in the evaluation. The calculated distance value $d_{M}$ denotes the overall goodness score for the quality of the segmentation result, *s*. Therefore, the performance of the segmentation algorithms or the parameter optimizations can be compared by finding a quality point that represents the segmentation result with the furthest distance to point (1,0) in the MI−q space.

### 2.4. Other Evaluation Measures

This paper presents three existing segmentation evaluation methods for comparison. Two of the methods are composite unsupervised measures and follow the characteristic criteria that include two metrics to measure the intra-segment uniformity and inter-segment disparity. These methods, and how they combine the two metrics, are summarized in Table 1. In addition, a supervised method is included to validate the effectiveness of the proposed method and to provide a direct and objective comparison between segmented images and reference images.

The first method is an entropy-based evaluation method proposed by Zhang et al. [44]. This method is based on information theory and the minimum description length (MDL) principle. The method uses entropy as the basis for measuring the uniformity of the pixel characteristics within a segment. An indicator, *E*, is built, which additively combines both the layout entropy $H_{l}$ and the expected region entropy $H_{r}$ when measuring the effectiveness of a segmentation method:

(7) $$H_{r}\left(I\right)=\overset{L}{\underset{h=1}{\sum }}\left(\frac{N_{h}}{N}\right)\left(-\underset{m\epsilon V_{h}}{\sum }\frac{L_{h}\left(m\right)}{N_{h}}log\frac{L_{h}\left(m\right)}{N_{h}}\right),$$

(8) $$H_{l}\left(I\right)=-\overset{L}{\underset{h=1}{\sum }}\frac{N_{h}}{N}log\frac{N_{h}}{N_{h}},\;$$

(9) $$E=H_{l}+H_{r},\;$$

where the meanings of the variable symbols are equivalent to those in Equation (4), and $L_{h}\left(m\right)$ denotes the number of pixels in segment h that have a value of m for intensity. $H_{r}$ reflects the intra-segment homogeneity by measuring the entropy of the pixel intensities within each segment. $H_{l}$ is used to prevent over-segmentation when $H_{r}$ becomes too small.

The second measure is the so-called Z method proposed by Zhang et al. [34]. Two metrics are included in Z, the homogeneity part *T* and the heterogeneity part *D*, where the former is modified from the Q method proposed by Borsotti et al. [45]:

(10) $$T\left(I\right)=\frac{1}{10N}\sqrt{L}\overset{L}{\underset{h=1}{\sum }}\frac{e_{h}^{2}}{1+\log N_{h}},\;$$

(11) $$D\left(I\right)=\frac{\sum_{b=1}^{B}\sum_{h}^{L}\frac{\left(m_{b_{h}}-mm_{b}\right)}{L}}{\sqrt{L}},$$

(12) Z=T+λD, 

where $e_{h}^{2}$ is the mean feature vector error, $m_{b_{h}}$ represents the mean spectral value of band b in segment *h*, $mm_{b}$ denotes the mean spectral value of band b in all segments, and B is the number of spectral bands. The weight is defined as: $\lambda =\left(T_{max}-T_{min}\right)/\left(D_{max}-D_{min}\right)$. For comparison purposes, the value of Z is reduced by a factor of 10,000 in this paper.

The Adjusted Rand Index (ARI) is a supervised evaluation method proposed by Huber and Arabie [46], which is a method for clusterings comparison [47]. As a pair counting-based measure, the ARI is built on counting pairs of items in which two clusterings or partitions agree or disagree. O is the segments set {$O_{1},\ldots ,O_{L}$} of the segmentation result of the testing image and $O^{\prime }$ = {$O_{1}^{\prime },\ldots ,O_{R}^{\prime }$} denotes the segments set of the reference image. N is the number of pixels. The segment sets can be summarized in a contingency table, M, where each entry $m_{ij}$ denotes the number of intersecting pixels between O and $O^{\prime }$: $m_{ij}$ = $\left|O_{i}\cap O_{j}^{\prime }\right|$. The ARI of O and $O^{\prime }$ is defined as follows:

(13) $$ARI\left(O,O^{\prime }\right)=\frac{\sum_{i=1}^{L}\sum_{j=1}^{R}\left(\begin{array}{c} m_{ij}\\ 2 \end{array}\right)-t_{3}}{\frac{1}{2}\left(t_{1}+t_{2}\right)-t_{3}},$$

where

(14) $$t_{1}=\overset{L}{\underset{i=1}{\sum }}\left(\begin{array}{c} card\left(O_{i}\right)\\ 2 \end{array}\right),\;t_{2}=\overset{R}{\underset{i=1}{\sum }}\left(\begin{array}{c} card\left(O_{j}^{\prime }\right)\\ 2 \end{array}\right),t_{3}=\frac{2t_{1}t_{2}}{N\left(N-1\right)},$$

The notations $card()\;and\;\left(\;\overset{\cdot }{\underset{\cdot }{\;}}\;\right)$ denote the cardinality operator and combinatorial notation, respectively. ARI has an upper bound of 1, indicating a perfect agreement between the reference and the segmented result. A large ARI value indicates a high correspondence to the reference.

### 2.5. Segmentation Methods

This paper implements the evaluation methods $d_{M},Z$, *E* and ARI on the segmentation results from two segmentation algorithms to compare both the various parameterizations of one segmentation method as well as fundamentally different segmentation techniques.

The multi-resolution segmentation (MRS) algorithm is based on the fractal net evolution approach (FNEA) [48]. The MRS is a bottom-up region-merging technique based on local criteria and begins with one pixel of an image object. The adjacent image objects are merged one by one in a pairwise manner to form a larger object. The underlying optimization procedure minimizes the weighted heterogeneity, which includes the color and shape heterogeneity. The shape heterogeneity describes the changes of compact degree and smooth degree before and after two adjacent regions are merged. The “scale” parameter is defined to satisfy the direct relationship between the image scale and the object size, which is the stop criterion for the optimization process when the smallest growth exceeds the defined threshold. Namely, the larger the scale is, the larger the segment size is.

Mean-shift segmentation (MSS) is a robust and adaptive clustering algorithm that uses non-parametric density estimation [49]. MSS shifts the original image data points in the feature space to the local maxima of the density function using a certain number of iterations and subsequent clustering of the filtered data points. After clustering, the filtered points are converted into a segment. This segmentation algorithm has three parameters, spatial bandwidth, color bandwidth and the Minimum Region. The “Minimum Region” parameter can be tuned to obtain the multi-scale segmentation results.

## 3. Experiments

### 3.1. Experimental Data

**Figure 4:**
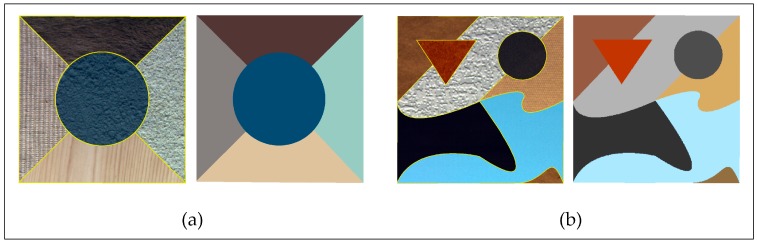
(**a**) The synthetic image S1 and its ground truth; and (**b**) the synthetic image S2 and its ground truth. The object boundaries of reference segmentation on original images are delineated by yellow lines.

To compare the difference between the feature set and original image used as input data for the proposed evaluation method, two synthetic images, named S1 and S2, respectively, were created for testing (Figure 4). These original images and ground truth were manually produced. The original images containing different textures are the simulation of the ground true objects. The textures used to create these images were randomly extracted from the Oulu’s University texture database (http://www.outex.oulu.fi).

The reason for using the synthetic image is that its ground truth is available, and the reference segmentation results conform to reality. Therefore, the experimental results can accurately reflect the difference between the original image and feature enhanced image by comparing them with the ground truth. In addition, the effectiveness of feature set is further validated by remote sensing images.

**Figure 5:**
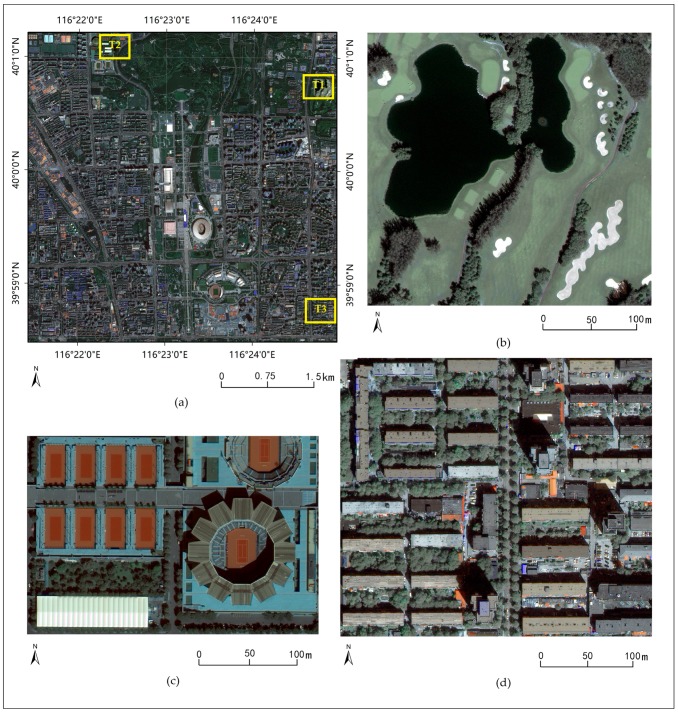
The WorldView-3 scene of study area (**a**); and subscenes: T1 (**b**); T2 (**c**); and T3 (**d**) used as the experimental data. The images are shown with a combination of red, green, and blue spectral bands.

To verify the effectiveness of the proposed method in remote sensing image segmentation, a study area was selected in the northern part of the Beijing central zone, China (39°58′29′′–40°1′13′′ N, 116°21′24′′–116°24′58′′ W). The study area includes abundant urban landscapes. A WorldView-3 scene of this area that was acquired on 3 September 2014 was used as the experimental data. The image contains information in the NIR, red, green, and blue spectral bands. The spatial resolution of the image was increased from 1.6 m to 0.4 m using the Gram-Schmidt pan-sharpening method [50] for a better recognition. In practices of GOBIA, the final segmentation result for a large-scale image is produced by multiple segmentation operations at different scales. In this paper, one result only produced by one segmentation step with a fixed parameter for verifying the effectiveness of the proposed method. Therefore, experiments were performed on three testing images with small scales, which are identified as T1, T2 and T3, and represent the landscapes of a watery area (540 × 520 pixels), a commercial area (534 × 487 pixels), and a residential area (594 × 530 pixels), respectively (Figure 5).

### 3.2. Experimental Setting

**Figure 6:**
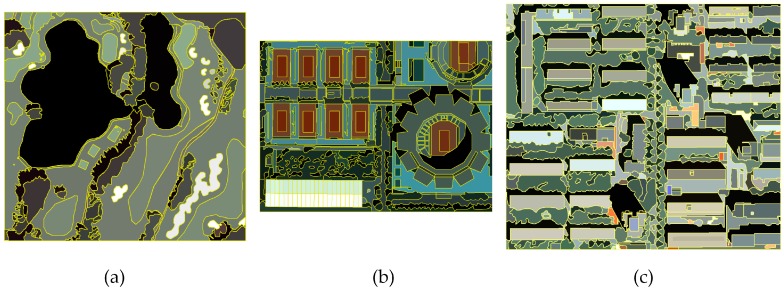
The ground truth of subscenes: T1 (**a**); T2 (**b**); and T3 (**c**). The reference segmentation boundaries are delineated by yellow lines. The values of objects in the ground truth are the mean value of the reference segments of original images.

In the experiments of testing the effectiveness of feature set, three types of base images were involved: original images, feature enhanced images and the ground truth. Reference segmentation boundaries were overlapped on these base images where the indictor q and MI of segments and the combined metric $d_{M}$ were computed, respectively. For each band of the testing images, the parameters of bilateral filter were set as $\sigma_{s}=3$ and $\sigma_{r}=0.1$. All single band filtering results were stacked into a spectral feature set. Two frequencies (v = 1, 2) and eight orientations (u = 2, 4, 6, 8, 10, 12, 14, 16; U = 8) composing a total of 16 Gabor kernels were chosen in experiments to make clearer distinctions among the different textures. That is, 16 spatial feature images were captured. Principal component analysis (PCA) was used on these spatial feature images to extract the top three principal components to reduce redundancy. The final feature enhanced image consisted of spatial features and spectral features with equal weights. The ground truth of each image was created by the real form of objects directly (synthetic images, Figure 4) or the reference segmentation results (remote sensing images, Figure 6), manually produced by a remote sensing expert.

In the experiments of verifying the effectiveness of the proposed metric $d_{M}$, multi-scale segmentation results from over- to under-segmentation for evaluation were produced using the MSS and MRS algorithms with different parameters. Due to the varying implications, the scale parameters of both algorithms were set and unified to a serial of scale levels to achieve comparable segmentation scales. For example, the corresponding scale levels for the testing image T3 are presented in Table 2, in which p represents the scale parameter and L is the number of generated segments with this scale parameter. The scale parameter of MSS is dynamic and changes with the different testing images to keep the number of segments similar to those of MRS at the same scale levels. Additionally, the following fixed parameters were used during the experiments: for MRS, the parameter color/shape was set to 0.1 and the smoothness/compactness to 0.5; for MSS, the parameter spatial bandwidth was set to 7, and the color bandwidth to 6.5. The segmentation boundaries were overlapped on this feature set as the base images of the proposed evaluation method. As discussed above, the reference images for the supervised method, ARI, were manually produced (Figure 6). The other evaluation methods described in Section 2.4 were also performed to evaluate and compare the segmentation results of T1, T2 and T3.

### 3.3. Results and Analysis

#### 3.3.1. Effectiveness of the Feature Set

Based on the original images, feature enhanced images and ground truth, the proposed evaluation method was performed on the reference segmentation of testing images. The evaluation results can be seen in the Table 3 and Table 4.

As indicated by Table 3, it is obvious that using ground truth of synthetic images as the base images to evaluate reference segmentation leads to the optimal result, which can be seen as a benchmark to assess the performance of the original image and the feature enhanced image during the evaluation process. The values of $d_{M}$ demonstrate that the evaluation results computed from the feature enhanced image are superior to those from the original image, and closer to the ground truth. Similarly, the ground truth of remote sensing images originates from interpretation of experts, further proves that the feature enhanced image greatly improves the evaluation metrics (Table 4). Specifically, this superiority mainly benefits from the significant improvement of q value of testing images in the feature enhanced image case. The spatial stratified heterogeneity of the original image is enhanced by feature extraction. In comparison, MI is less sensitive to the base image, and there are no prominent differences between the values of MI computed from original image and feature enhanced image. However, the variation of the combined metric $d_{M}$ for the reference segmentation result indicates that the feature set composed of spectral and spatial features can describe the form of the real objects more accurately than the original images. Therefore, the proposed method based on the feature enhanced images yields evaluation results that are more consistent with reality.

#### 3.3.2. Effectiveness of q-Statistics and MI Metric

**Figure 7:**
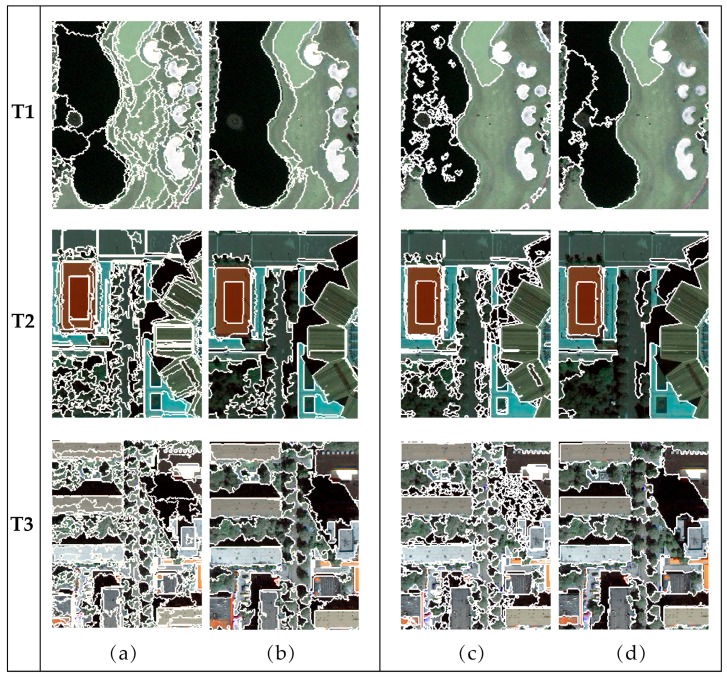
Subsets of multiscale segmentations produced by MRS and MSS for testing images T1, T2 and T3: (**a**,**b**) the results using the MRS algorithm at scale 2 and scale 19, respectively; and (**c**,**d**) the results using the MSS algorithm at scale 2 and scale 19, respectively. The segmentation boundaries are delineated by white lines.

Both MRS and MSS segmentation were performed at 20 scales, ranging from level 1 (over-segmentation) to 20 (under-segmentation) to assess the effectiveness of the proposed measure across the remote sensing images. Two segmentation results of each testing image, corresponding to scale 2 and scale 19, are displayed in Figure 7. In Figure 7a,c, the images are significantly over-segmented at scale 2, and some areas are too fragile to sketch the outline of an integral object. In Figure 7b,d, however, the segmentation results at scale 19 evidently exhibit under-segmentation. The sizes of the segments are too large, and the boundaries of some objects are not precisely delineated, thus leading to mixed land cover in one segment. As shown in Table 2, the number of segments in these results decreases as the scale level increases. The optimal result is between the over- and under-segmentation. A quantitative method is required to select the best scale parameter.

**Figure 8:**
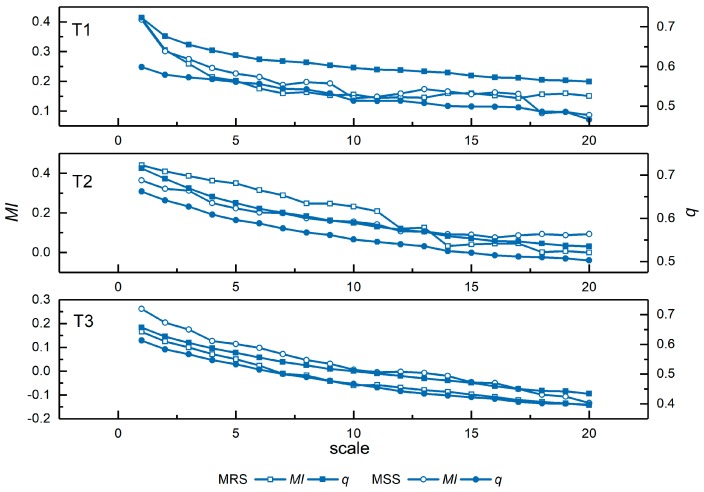
Changes in q and MI  from the segmentation results of T1, T2 and T3 using MRS and MSS at scales 1 to 20.

The changes in the q and MI values of the segmentation results using MRS and MSS from scales 1 to 20 are shown in Figure 8. As the scale level increases, both q and MI tend to decrease, which means that the values of q and MI are higher when the result is over-segmented than when it is under-segmented. As discussed in Section 2.2, the q-statistic indicates the degree of spatial stratified heterogeneity, which is sensitive to the variance both within- and between-strata; MI is a spatial autocorrelation metric that indicates the global inter-segment goodness. In the over-segmented case, the average size of segments is small and the intra-segment homogeneity is high. Adjacent segments have a greater degree of similarity in an over-segmented case than in an under-segmented case. Thus, the spatial autocorrelation of the segmentation result mainly reflects the inter-segment heterogeneity, which is low, and, accordingly, the values of MI and q are high with respect to under-segmentation. As the scale increases, the average size of the segments increases, the segments tend to include more dissimilar pixels, and neighboring segments become less similar to one another. Therefore, the intra-segment homogeneity decreases, whereas the between-segment heterogeneity increases, leading to a decrease of both q and MI. Figure 8 shows that these two indicators, q and MI, appropriately reflect the variation at different segmentation scales for both the MRS and MSS methods.

**Figure 9:**
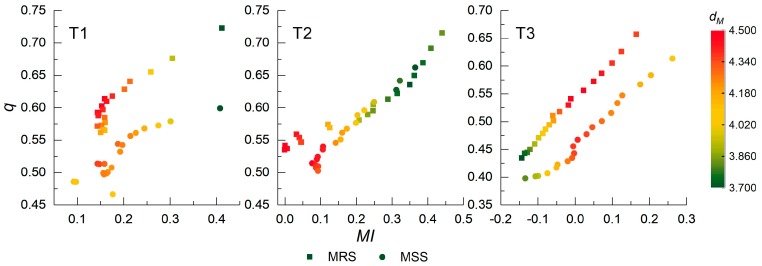
Quality points consisting of MI and q  variables in the two-dimensional MI−q  space of T1, T2, and T3. Each point corresponds to a segmentation result of MRS or MSS, and colors indicate the Mahalanobis distance to the quality point (1,0).

The q and MI values were combined into the MI− q space as the quality points, as shown in Figure 9. As discussed in Section 2.3, each point corresponds to a segmentation result, marked by a certain color indicating the Mahalanobis distance to point (1,0). From the distribution of these points in Figure 9, the point with the largest value of $d_{M}$ satisfies the pairwise constraints of the highest q and the lowest |MI| value. In addition, the points with both high (over-segmented) and low (under-segmented) values of q and MI result in small $d_{M}\;$values. For example, in Figure 9, the points (0.16,0.61) and (0.14,0.51) in T1 indicate the largest $d_{M}\;$values of MRS and MSS, respectively, whereas the points (0.41,0.72) and (0.41,0.60) correspond to the smallest $d_{M}\;$values. Similarly, it is easy to locate the best segmentation results for testing images T2 and T3 in Figure 9. The combination strategy of these two indicators can quantify the dissimilarity and reveal the quality of the corresponding segmentation results directly. The details of identifying the optimal parameters or segmentation method using the indicator $d_{M}\;$will be discussed in the next section.

#### 3.3.3. Effectiveness of the $d_{M}$ Metric

**Figure 10:**
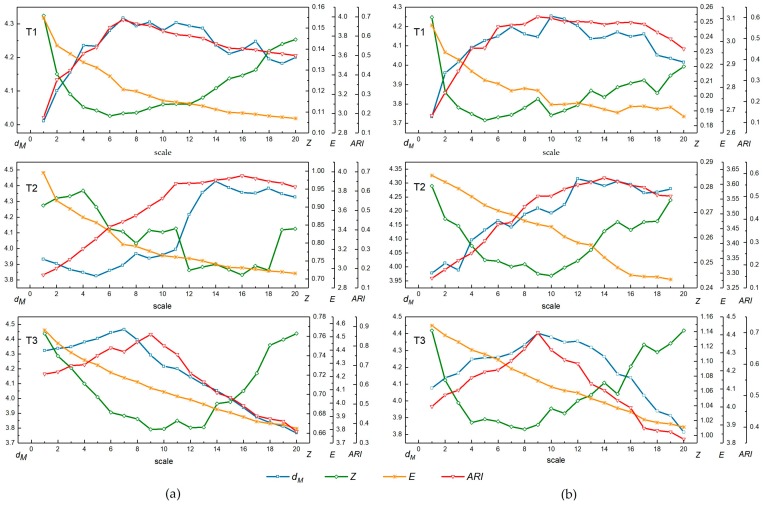
Comparing the $d_{M}$ method with existing evaluation methods (Z, E and ARI) for testing images T1, T2, and T3: (**a**) plots of evaluation methods for MRS with different scale parameters; and (**b**) plots of evaluation methods for MSS with different scale parameters.

The combined indicators were further evaluated by comparing existing evaluation methods to assess the effectiveness of the proposed method, as presented in Section 2.4. The variation trends of the unsupervised methods $d_{M}$, Z, and E and the supervised method ARI for the testing images are plotted in Figure 10 at different scales. Figure 10a is for MRS and Figure 10b is for MSS. As defined in Section 2.4, larger $d_{M}\;$and ARI values and smaller Z and E values indicate superior segmentation quality.

Specifically, for T1 and T3, the value of $d_{M}$ increases significantly at small scales and then decreases as the scale becomes large. The $d_{M}$ value is maximized in T1 at scales 7 and 10 for the MRS and MSS algorithms, respectively, and it is maximized in T3 at scales 7 and 9 for the MRS and MSS methods, respectively, which means that the segmentation is optimal at these scales. In addition, it can be seen in Figure 10a,b that $d_{M}$ for T1 has a high growth rate at the initial stages, and the decline rate is relatively slow at the larger scales after the maximum is reached. Conversely, the growth rate of $d_{M}\;$for T3 at small scales is slow, but it decreases sharply after reaching the maximum. The graphs show different trends for T1 and T3 because different landscapes are included in the two images. As seen from Figure 7, the patterns are rather simple in T1, including a large area of water, grassland and golf courses, all of which are homogeneous. However, T3 includes a dense residential area that contains many categories of objects such as roofs, tree crowns, shadows, and cars. The average sizes of these objects are smaller than those in T1. Therefore, T1 is prone to over-segmentation at small scales, and the segmentation becomes steady at large scales. T3 is more sensitive to under-segmentation at large scales due to the small size objects, and the method is well suited at small scales. For T2, an anomalously continuous decrease of $d_{M}$ occurs from scale 1 to 5 for MRS, and the value of $d_{M}$ increase in waves as the scale increases for both segmentation algorithms. The reason for this phenomenon is that the average size of the objects in T2 is large, while the internal heterogeneity is high. However, the overall trend can still correctly reflect the change of segmentation parameters, and indicates optimum scales are 14 and 12 using the MRS and MSS methods, respectively.

The variation tendency of Z is opposite to that of $d_{M}\;$across scales. For both the MRS and MSS algorithms, Z decreases gradually at small scales, reaching the minimum at scales 6 and 5, respectively, for T1 and at scales 9 and 8, respectively, for T3 and then continues to increase as the scale increases. For T2, Z appears to undulate initially but continues to follow the general trend of increasing after decreasing, reaching the minimum at scales 16 and 10, respectively. E continuously decreases as the scale increases for all testing images using both segmentation algorithms. E indicates that the optimal segmentation occurs when the scale is large. In general, $d_{M}\;$ and Z respond to the segmentation results well and clearly reflect the change from over- to under-segmentation. However, E is a monotonic index that ignores the influence of the quality from under-segmentation. The optimal scales indicated by these three unsupervised evaluation measures are different from each other.

**Figure 11:**
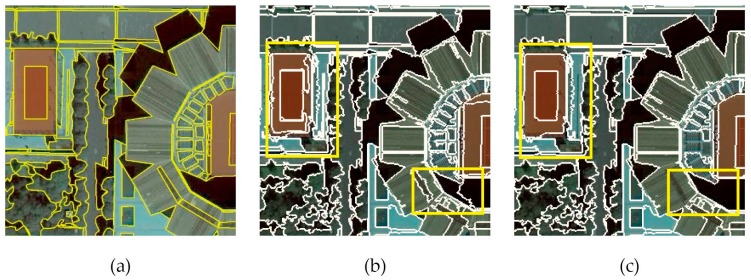
(**a**) Subset of the reference results for T2; (**b**) subset of segmentation results produced by MRS for T2 at scale 14; and (**c**) subset of segmentation results produced by MRS for T2 at scale 16. The main differences between (**b**) and (**c**) are highlighted by the yellow rectangles. The object boundaries of the reference image are delineated by yellow lines.

To quantitatively compare the performance of $d_{M},\;Z,\;\mathrm{and}\;E$, the Spearman’s rank correlation coefficient was computed between every unsupervised method and ARI. A perfect Spearman correlation of 1 or −1 occurs when each of the variables is a monotonic function of the other. The results are shown in Table 5. Though the Z method performs poorly on the segmentation results of MSS for T1 and T2, it still yields a high correlation with ARI in MRS case. As exhibited in Figure 10, the method E behavesmonotonically as the segmentation scale increases, and its correlation coefficient is unstable. Obviously, the method $d_{M}$ consistently yields higher correlations with the supervised method ARI than the other methods for both MRS and MSS. The general trend of $d_{M}$ in Figure 10 is similar to that of ARI for all testing images. For example, using MRS, the ARI continuously increases from scales 1 to 16 for T2 and then gradually decreases for scales larger than 16. As mentioned above, the overall trend of $d_{M}$ increases with the scale until a scale of 14. Figure 11b,c display a subset of the results at the optimal scales given by $d_{M}$ and ARI, respectively. Specifically, the value of $d_{M}\;$ is 4.427 and ARI is 0.656 at scale 14; the value of $d_{M}$ is 4.356 and ARI is 0.679 at scale 16. Figure 11a shows a subset of the reference image for T2. The optimal segmentation results indicated by $d_{M}$ and ARI are similar, and both can adequately discriminate between geographic objects. The yellow rectangles in Figure 11b,c mark some minor differences.

**Figure 12:**
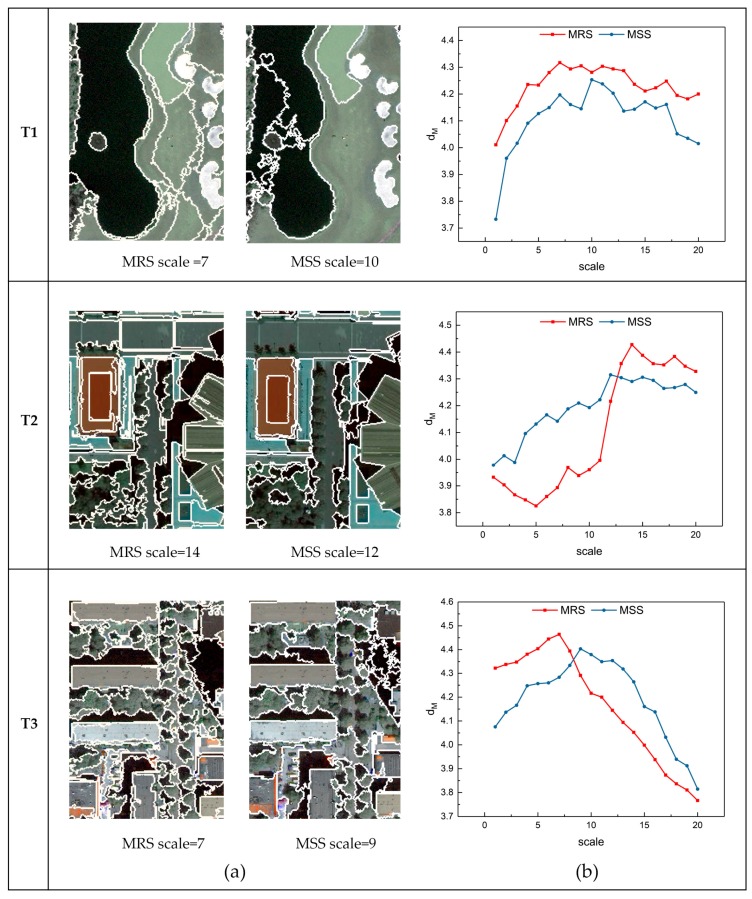
(**a**) The optimal segmentation results of MRS and MSS indicated by the $d_{M}$ method for T1, T2 and T3; and (**b**) the plots of the $d_{M}\;$ values for MRS and MSS at different scales.

In addition to determining the optimal parameters, the $d_{M}$ method can be useful for the comparison of segmentation algorithms. Considering the scale parameter of the segmentation, Figure 12 illustrates that the $d_{M}$ value of the optimal segmentation quality using MRS is higher than that using MSS for all three testing images. However, the performance between MRS and MSS differs at different scales for different testing images. For T1, the $d_{M}$ values of MRS are higher than those of MSS at all 20 scales, indicating that the performance of MRS is superior to MSS in T1 from over- to under-segmentation. The $d_{M}$ values indicate that MSS performed better than MRS at scales 1 to 12 for T2, and at scales 9 to 20 for T3.

Visual analysis was applied for further comparison of the two segmentation methods based on $d_{M}$. Figure 7 and Figure 11 illustrate a subset of the segmentation results for the testing images at small, large, and optimal scales. The results in T1 (Figure 7) indicate that the MSS cannot distinguish grassland types with different textures, even at small scales. Moreover, the performances of MSS on extracting low-intensity objects, such as water and shadows, are poor (typical parts are shown in Figure 7c,d). T1 contains large areas of water and grassland (Figure 5b), leading to the inferior quality of the results generated from MSS in comparison to those of MRS, which is consistent with the results indicated by $d_{M}$. At small scales, MSS is able to completely delineate the large roof of a stadium in T2 and ignores the noise within the roof (Figure 7c), which is superior to MRS even at optimal scales. Therefore, MSS outperforms MRS at scales 1 to 11 in an over-segmented case. However, the performance of MSS is constrained at larger scales since it cannot distinguish tree crowns from streets (Figure 7d). The objects in T3 are smaller than those in T1 and T2, so MRS and MSS result in high $d_{M}\;$values at small scales. The shadows of the buildings are segmented into fragmentized regions using MSS at small scales. The roofs in T3 (Figure 7b) are not segmented accurately using MRS at large scales. The under-segmentation of MRS at large segmentation scales for T3 is inferior to that of T1 and T2 when the scales are smaller. The visual analysis is reflected well by the $d_{M}\;$values in Figure 12.

## 4. Discussion

Several issues need to be further discussed.

(1) In the experiments, q and MI were calculated from the spectral-spatial feature set rather than from the raw images. The evaluation of testing images with reference segmentation indicates that the feature enhanced images are superior to the original images for describing real objects. The optimal results indicated by $d_{M}\;$are more likely to approach the results desired by evaluators. However, if only the original spectral features of the remote sensing images containing complicated land cover are used for evaluation, the assessment criterion may not be sufficient to indicate the segmentation quality accurately in terms of the real forms of the objects, and the optimal parameter and segmentation result selected by $d_{M}\;$may be inconsistent with a visual perception of the objects. The spectral features extracted using the bilateral filter help evaluate the indicator while ignoring slight intensity variations within the objects. The spatial features extracted using the Gabor filter provides complementary edge and texture information. In practical applications, the optimal segmentation result selected using a spectral-spatial feature set is more consistent with the spatial distribution of the landscapes in the real world, as discussed in Section 3.3. It is impractical to attempt to obtain exact ground truth of remote sensing images during segmentation evaluation. The use of spectral-spatial features can overcome some disadvantages of unsupervised measures without using a reference image.
(2) As a significant indicator in geostatistics, the introduced spatial stratified heterogeneity can successfully indicate the intra-segmentation property, as demonstrated by Figure 8. The spatial autocorrelation metric MI is able to indicate the inter-segmentation dissimilarity. Both indicators are sensitive to the variation of the segmentation results for all testing images, and they supplement each other for segmentation evaluation. These two indicators are combined to reveal the segmentation quality by achieving the balance between them. The proposed combination strategy using the Mahalanobis distance between quality points, which is calculated by the | MI| and q values and the point (1,0) in the MI−q space, is an effective criterion that can be intuitively visualized by geometric illustration. The position of each quality point in MI−q space represents a certain state of segmentation, which can be seen in Figure 9. In the case of over-segmentation, both | MI| and q are large, and the corresponding quality point is located in the upper-right portion of the MI−q space. In contrast, both small | MI| and q values indicate under-segmentation and the quality point is located in the bottom-left portion of the MI−q space.
(3) The effectiveness of the $d_{M}\;$metric was confirmed by conducting experiments to determine optimal parameter selection and compare different segmentation algorithms. Relative to other existing unsupervised evaluations, $d_{M}\;$illustrates the segmentation results more clearly. Specifically, E cannot respond to change in scales from over- to under-segmentation, and the sensitivity and response of Z to different segmentations is similar to $d_{M}$. The selection of optimal segmentation is slightly different. Rank correlation analysis quantitatively indicates that $d_{M}\;$achieves results closer to the supervised method ARI, which is relatively reliable compared to unsupervised measures. Moreover, the visual analysis in Section 3.3 further confirms the effectiveness of $d_{M}$ in comparison with other segmentation algorithms. The relationship between the characteristics of multiple testing images and the segmentation algorithms with different parameters is presented clearly by the variation of $d_{M}$, and facilitates the next steps of the analysis, such as classification and object detection.
(4) The main factor that affects an evaluation method’s use of computational resources is the number of segments. Analyzing each evaluation method with an input-data size *n*, the computational complexity described using big O notion is O(n) for method E and O$\left(n^{2}\right)$ for methods Z and ARI. For method $d_{M},$ because matrix operations are involved in calculating the Mahalanobis distance, the computational complexity initially appears higher than for other methods. However, the dimensions of the matrix depend on the number of segmentation results involved in evaluation, which is generally not within the same order of magnitude as the number of segments. Therefore, the influence of matrix operations in $\mathrm{method}\;d_{M}$ can often be ignored, resulting in a computational complexity similar to the method Z and ARI. However, when the number of segmentation results participating in the evaluation is no negligible compared with the number of segments, the computational complexity of $d_{M}$ is higher than that of the other methods because of the matrix operations. The Euclidean distance can then be used as a substitute in the combination strategy to reduce the computation load.

Future studies should address the following aspects:

(1) Although the spectral-spatial feature used in the proposed method is advantageous for representing real objects, the method of constructing feature models and the choice of extractors still could be optimized. In addition, the strategy used to stack the spectral and spatial features involves simple combination. A more sophisticated strategy could be developed that might extract features more efficiently and accurately.
(2) Scale is important for the interpretation of remote sensing images and is an important parameter of the segmentation algorithms. This paper only focuses on the optimal choice of the scale parameter in segmentation quality evaluation. The universality and robustness of the proposed method should be verified for the other segmentation parameters.
(3) The experiment only tested three sub-images from the same WorldView-3 scene. However, in practices of GEOBIA, it is necessary to evaluate the integrated image segmentation results, which may consume a large amount of computing resources. Thus, to increase efficiency, small and randomly selected sub-images could be used as samples to evaluate the segmentation results of a large-scale image.

## 5. Conclusions

A novel unsupervised method is proposed for evaluating the segmentation quality of VHR remote sensing images. This method uses a multidimensional spectral–spatial feature set as the feature image, which is captured from a raw image using a bilateral filter and a Gabor wavelet filter. Based on this integrated feature set, q and MI, which respectively denote the spatial stratified heterogeneity and spatial autocorrelation, are computed to indicate the property of each segmentation result from different aspects. These two indicators are then combined into a single overall metric $d_{M}$ using a strategy of measuring the Mahalanobis distance of the quality points in the MI−q space to reveal the segmentation quality. Evaluations of reference segmentation of two synthetic images and three remote sensing images indicate that applying the proposed method to a feature enhanced image yields superior results relative to the original image. The MRS and MSS segmentation algorithms with different parameters were applied to the three remote sensing images to produce multiple segmentation results for evaluation. The experimental results show that indicators q  and MI appropriately reflect the changes at different segmentation scales, and the combined metric $d_{M}$ clearly reveals the segmentation quality when applied to different algorithms and different parameters. The effectiveness of the combined metric, $d_{M},$ is further demonstrated by comparing two existing unsupervised measures and one supervised method. The results demonstrate the superior potential and robust performance of the proposed method.

## Figures and Tables

**Table 1:** The measures used in the existing and proposed unsupervised evaluation methods

Name	Intra-Segment	Inter-Segment	Intra-and Inter-Segment
	Metrics	Metrics	Combination
*E*	Entropy	Entropy	Sum
Z	Squared color error	Heterogeneity	Weighted sum
$d_{M}$	Spatial stratified heterogeneity	Spatial autocorrelation	Mahalanobis distance

**Table 2:** Scale levels of MRS and MSS for testing image T3. *p* represents the scale parameter, and *L* represents the corresponding number of segments in the segmentation result.

Scale	MRS	MSS	Scale	MRS	MSS
	*p/L*	*p/L*		*p/L*	*p/L*
1	25/1826	30/1846	11	75/257	345/255
2	30/1274	46/1270	12	80/237	389/234
3	35/968	65/974	13	85/215	418/214
4	40/763	90/762	14	90/193	500/193
5	45/625	117/626	15	95/177	560/175
6	50/510	155/508	16	100/154	700/154
7	55/424	188/424	17	105/137	800/136
8	60/370	220/369	18	110/127	900/126
9	65/321	264/320	19	115/124	920/123
10	70/289	305/287	20	120/114	1100/114

**Table 3:** Values of the evaluation metric on the reference segmentation results of S1 and S2.

Base Image	q	MI	$d_{M}$
	S1/S2	S1/S2	S1/S2
Original image	0.901/0.873	0.011/0.366	2.480/2.175
Feature enhanced image	0.926/0.943	0.011/0.351	2.512/2.308
Ground truth	1.000/1.000	0.010/0.043	2.601/2.808

**Table 4:** Values of the evaluation metric on the reference segmentation results of T1, T2 and T3.

Base Image	q	MI	$d_{M}$
	T1/T2/T3	T1/T2/T3	T1/T2/T3
Original image	0.784/0.610/0.701	0.301/0.259/0.159	2.218/1.975/2.230
Feature enhanced image	0.806/0.639/0.726	0.300/0.234/0.132	2.252/2.009/2.305
Ground truth	1.000/1.000/1.000	0.191/−0.001/0.076	2.710/2.907/2.775

**Table 5:** The value of the Spearman’s rank correlation between each unsupervised method and the supervised method *ARI*. Respectively, $\rho_{\mathrm{MRS}}$ and $\rho_{\mathrm{MSS}}$ indicate the correlation coefficient ρ from the evaluation results for MRS and MSS.

Testing Image	$d_{M}$	Z	E
	$\rho_{MRS}/\rho_{MSS}$	$\rho_{MRS}/\rho_{MSS}$	$\rho_{MRS}/\rho_{MSS}$
T1	0.926/0.821	−0.806/−0.196	0.008/−0.376
T2	0.872/0.934	−0.827/−0.152	−0.816/−0.737
T3	0.833/0.923	−0.677/0.898	0.681/0.498
